# 

**Published:** 1904-05-28

**Authors:** 


**NOTICE TO CORRESPONDENTS.**

All MSS., Letters, Books for Review, and other matters intended for the Editor should be addressed THE EDITOR, "THE HOSPITAL," THE HOSPITAL BUILDINGS, 28 & 29 SOUTHAMPTON STREET, STRAND, W.C.

The Editor cannot undertake to return rejected MS., even when accompanied by stamped directed envelope.

**Notice to Advertisers and Subscribers.**

All Advertisements, Orders for Copies of THE HOSPITAL, and Business Communications should be addressed to **THE MANAGER (Not to the Editor),** THE HOSPITAL, 28 & 29 Southampton Street, Strand, London, W.C.

The Cost of Subscription, post free, is as follows:—Per week, 3½d.; per quarter, 3s. 3d.; per half-year, 6s. 6d.; per annum, 13s. Foreign and Colonial:—Per annum, 19s. 6d., payable, in all cases, in advance.

**NOTICE.—Vol. XXXV. of "The Hospital" (October 1903 to March 1904), in handsome cloth binding, is now on sale at the office of this Journal, price 10s. 6d. Cases for binding the Numbers contained in this Volume 2s. Index to Vol. XXXV., 3½d., post free.**

**The Monthly part for June will be ready on June 1. Price 1s., by post, 1s. 3d.**

A NEW operating theatre has been opened at the Yeovil Hospital at a cost of £1,000.

THE Lord Lieutenant of Ireland opened last week a Home for Nurses at the Cork Street Fever Hospital, Dublin. The expenditure has been met by a legacy of the late Mr. James Weir; and the Home will bear his name.

A NURSING HOME for paying patients has been opened at Ipswich. It will be supervised by the Matron of the Ipswich Nurses' Home. The fees will range from £2 2s., and should any profit accrue, it will be devoted to the nursing of the sick poor. An operating-room and all nececessary appliances have been provided.

AT the annual meeting of the National Sanatorium, Bournemouth, it was announced that plans for the extension of the buildings had been prepared. New quarters for nurses, and further accommodation for patients will be provided. One thousand pounds is still needed to defray the cost of the improvements recently completed.

A new out-patient's department has been opened at the Western Ophthalmic Hospital, Marylebone Road. The cost has been £3,000.

### MEDICAL APPOINTMENTS.

**Arthur Bevan**, M.D.Lond., M.R.C.S.Eng., L.R.C.P.Lond., Anæsthetist to St. Thomas's Hospital. **William Frederick Cockaigne Bennett**, L.R.C.P.Edin., L.R.C.S.Edin., L.F.P.S. Glasg., L.S.A.Lond., District Medical Officer and Public Vaccinator, Sheffield Union. **A. T. F. Brown**, M.D.Durh, M.R.C S.Eng., Certifying Factory Surgeon for the Chatham District, Kent. **A. Douglas Cowburn**, M.R.C.S., L.R.C.P.Lond., D.P.H., Medical Officer of Health to the Honourable Societies of the Inner and Middle Temple. **L. Erasmus Ellis**, M.D.Brux., M.R.C.S. Eng., L.R.C.P.Lond., Clinical Assistant to the East London Hospital for Children, Shadwell, E. **J. Graham Forbes**, M.D. Cantab., M.R.C.P.Lond., Honorary Physician to the Farringdon General Dispensary. **H. Morgan Gilmour**, M.R.C.S.Eng., L.R.C.P.Lond., House Surgeon to the Bridgnorth and South Shropshire Infirmary. **T. MacMaster Glen**, L.R.C.P.&S.Edin., L.F.P.S.Glasg., Certifying Factory Surgeon for the Cauldbeck District, Cumberland. **P. W. Hamond**, M.R.C.S., L.R.C.P.Lond., Resident Assistant Medical Officer at the Lewisham Union Infirmary. **John Highet**, M.D., D.P.H., Medical Officer to Post Office at Troon, Ayrshire, including Dundonald and Lymington. **Philip E. Hill**, M.R.C.S, L.S.A., Medical Officer to the Post Office, Crickhowell. **G. Hodge**, M.B., C.M.Glasg., Assistant Physician to the Dumfries and Galloway Royal Infirmary. **W. E. L. Horner**, M.D., B.S.Lond., M.R.C.S., L.R.C.P., Medical Officer of Health to the United Urban District of Wolstanton. **J. Johnston**, M.D.Edin., L.S.A.Lond., Medical Officer of the Casual Wards of the Bolton Union. **A. J. Kennedy**, L.S.A., Certifying Factory Surgeon for the Shepley District, Yorkshire. **D. A. MacGregor**, M.B., C.M.Edin., Certifying Factory Surgeon for the Denby Dale District, Yorkshire. **G. E. Middlemist**, M.B., District Medical Officer of the Caistor Union. **Robert Orr**, M.B., Ch.B.Glasg., Medical Officer to the Parish of Ceres, and Certifying Factory Surgeon for the Civil District of Ceres, Fifeshire, N.B. **J. Herbert Parsons**, B.S., D.Sc., F.R.C.S., Assistant Ophthalmic Surgeon to University College Hospital. **J. Reid**, M.B., C.M.Aberd., Certifying Factory Surgeon for the Portsoy District, Banffshire. **T. Roberts**, M.B., C.M.Edin., Medical Officer to the Carnarvon Port Sanitary Authority and Isolation Hospital. **W. Robertshaw**, M.B., C.M.Edin., Certifying Surgeon for the Stocksbridge District, Yorkshire. **R. Douglas Sproat**, L.R.C.P., M.R.C.S., Resident Surgical Officer to the Children's Hospital, Birmingham. **T. W. Tetley**, M.R C.S., L.R.C.P., Medical Officer of Health for the Kirkby Moorside Rural District. **Chisholm Williams**, F.R.C.S.Edin., Surgeon to the City Orthopædic Hospital.

**"The Hospital" Institutional Library.**

"Hospitals and Asylums of the World." 4 Vols., with a Portfolio of Plans, complete £12 12s.
"Burdett's Hospitals and Charities, 1904." 5s. net.
"Burdett's Official Nursing Directory." 8s. net.
"Cottage Hospitals, General, Fever, and Convalescent." 10s. 6d.
"Uniform System of Accounts for Hospitals and Public Institutions." New Edition. 4s. net.
"Hospital Expenditure: The Commissariat." 2s. 6d.
"A Legal Handbook for the Use of Hospital Authorities." 2s. 6d.
"The Cottage Hospital Case Book or Register of Patients." 10s. and 12s. 6d.

All these are published by the SCIENTIFIC PRESS, Ltd., and may be obtained through any bookseller, or direct from the publishers 28 and 29 Southampton Street, Strand, London, W.C.

### Standard Nursing Manuals.

### A COMPLETE HANDBOOK of MIDWIFERY.

By J. K. Watson, M.D.Edin., Author of "A Handbook for Nurses."
Crown 8vo. over 350 pp., profusely illustrated with 143 Diagrams.
**6s.** net, **6s. 4d.** post free.

### A HANDBOOK FOR NURSES.

By J. K. Watson, M.D., M.B., C.M.
New and Revised Edition. Crown 8vo. profusely illustrated, cloth gilt.
**5s.** net, **5s. 4d.** post free.

### NURSING: Its Theory and Practice.

Being a complete Text-book of Medical, Surgical, and Monthly Nursing.
By Percy G. Lewis, M.D., M.R.C.S., L.S A.
New Edition, Enlarged and Revised (17th thousand), profusely illustrated with over 100 new Cuts. Crown 8vo. cloth gilt.
**3s. 6d.** post free.

### NURSING IN DISEASES OF THE THROAT, NOSE AND EAR.

By P. MacLeod Yearsley, F.R.C.S.Eng., M.R.C.S., L.R.C.P.Lond.
Demy 8vo. illustrated, cloth.
**2s. 6d.** post free.

### OPHTHALMIC NURSING.

By Sydney Stephenson, M.B., F.R.C.S.E.
New and Revised Edition. Crown 8vo. profusely illustrated with Original Drawings, list of Instruments required, and a Glossary of Terms, cloth.
**3s. 6d.** net, **3s. 10d.** post free.

### THE HUMAN BODY.

Its Personal Hygiene and Practical Physiology.
By B. P. Colton, Professor of Natural Science, Illinois University.
Crown 8vo. profusely illustrated, cloth gilt.
**5s.** post free.

### THE EXAMINATION OF THE URINE.

By J. K. Watson, M.D., M.B., C.M., Author of "A Handbook for Nurses."

Demy 16mo. cloth boards.

**1s.** net, **1s. 1d.** post free.

### SURGICAL WARD-WORK AND NURSING.

By Alexander Miles, M.D.Edin., C.M., F.R.C.S E.
Second Edition. Enlarged and entirely Revised. Demy 8vo. copiously illustrated, cloth gilt.
**3s. 6d.**, **3s. 10d.** post free.

### PRACTICAL GUIDE TO SURGICAL BANDAGING AND DRESSINGS.

By Wm. Johnson Smith, F.R.C.S.
Demy 16mo. profusely illustrated (suitable for apron pocket), cloth. **2s.** post free.

### ELEMENTARY ANATOMY AND SURGERY FOR NURSES.

By William McAdam Eccles, M.S.Lond., M.B., F.R.C.S., L.R.C.P.
Crown 8vo. profusely illustrated, cloth.
**2s. 6d.** post free.

### ELEMENTARY PHYSIOLOGY FOR NURSES.

By C. F. Marshall, M.D., B.Sc., F.R.C.S.
Crown 8vo. illustrated, cloth. **2s.** post free.

### OUTLINES OF INSANITY.

A Popular Treatise on the Salient Features of Insanity.
By Francis H. Walmsley, M.D.
Demy 8vo. cloth extra. **3s. 6d.** post free.
Popular Edition, stitched. **1s. 6d.** post free.

### ART OF MASSAGE.

By A. Creighton Hale.
Second Edition. Demy 8vo. 150 pp., profusely illustrated with over 70 special Cuts, cloth gilt. **6s.** post free.

### MEDICAL GYMNASTICS, Including the Schott (Nauheim) Movements.

Being a Text-book of Massage and Mechanical Therapeutics Generally.
By Axel V. Grafstrom, B.Sc., M D.
Crown 8vo. illustrated, cloth. **2s. 6d.**

### New Case Books.

### A CASE BOOK FOR PRIVATE NURSING.

Arranged for Day and Night Nurse's Report, with Rulings in accordance with what has been found in practice to be the most suitable and approved System for Pay Hospitals, Nursing Homes, Private Nurses, etc. The size of an Exercise Book, $7\frac{1}{2} \times 9\frac{1}{2}$ in., containing 48 pp. (Special quotations for a quantity.) **3d.** net, **4½d.** post free.

### THE "SIMPLEX" DISTRICT NURSE'S CASE BOOK.

Suitable size for the Pocket, containing space for 50 cases. Bound in cloth boards.
**6d.** net, **7½d.** post free.

### THE POCKET CASE BOOK FOR DISTRICT AND PRIVATE NURSES.

By a Physician.

Demy 16mo. cloth boards. **1s.**

In handsome leather, gilt, **1s. 6d.** net, **1s. 7d.** post free.

**Complete Catalogue post free on application.**

*Of all Booksellers or of*

THE SCIENTIFIC PRESS, LTD., 28 & 29 Southampton Street, Strand, LONDON, W.C.

